# Case Notes of an Obscure Disease

**Published:** 1915-09-18

**Authors:** 


### Case Notes of an Obscure Disease.

To take the cases as they come, the first is that of an English soldier, J. H., aged twenty-one, who came under treatment on April 11 complaining of pains all over, but chiefly in the back and legs, with malaise and weakness. These symptoms began about six or seven days before his admission to a base hospital. He felt first of all a slight pain in the regions named, while doing his tour of duty in trenches which had been dry for at least a month. Two days after being relieved in the trenches the pain became much worse, and the man states that his temperature was taken and was 103.2 deg. F. The invasion of the disease was therefore fairly rapid. The pain was not especially connected with the joints, though it was accentuated by movement. Beyond this, the patient complained of nothing. There had been no diarrhœa, and no nose bleeding. His previous medical history was quite unimportant, except that he had been inoculated against typhoid fever once, on January 1, 1915.

On admission, the man's temperature was 102.4 deg. F., and the pulse rate 84. The cheeks were rather flushed, but the man did not look very ill, and could turn easily in bed. The tongue was moist and very furred: herpes labialis was not present. There was no cough nor dyspnœa. There was no swelling of any joints, nor any œdema of the legs. Examination of the chest showed no signs of cardiac or pulmonary disorder. Unfortunately no record exists of the state of the reflexes and of the nervous system in general at this date: the case was regarded as acute rheumatism, and it was not until four or five days later, during which time salicylates had been very freely exhibited, that alternative suggestions were taken into consideration. During the period following the patient's admission to hospital a rash appeared for the first time, and it was this rash which drew attention to the unusual features of the case. The rash was distributed in abundance all over the body; but decidedly more on the extensor than on the flexor surfaces of the limbs, and more on the back of the trunk than on the front. It was present on the face and neck, and also on the hands and feet, even on the palms and soles. It came out in blotchy patches of erythema varying in size up to the surface of a split haricot bean, but mostly rather smaller than that. On their first appearance these patches of rash were often distinctly urticarial. The rash was very slightly raised, of a rather dull red colour; it disappeared on pressure, leaving a small amount of staining. The fully developed rash was somewhat morbilliform in type, except as to distribution. It appeared in a succession of copious crops, at intervals of two, three, or four days: each crop came out very rapidly, and sometimes before the last crop had completely faded, so that new and old spots were then present together. There were in all more than a dozen such eruptions, probably not less than fifteen or sixteen.

The temperature during this time rose to 102 or 103 deg. on most days, usually in the afternoon or evening: it was sometimes normal or sub-normal in the morning, but more often ranged about 100 deg. Headache developed during this second week of the disease, and constipation was a marked feature. The tongue remained plastered with a dense whitish-yellow fur, but was not raw or dry. Each day the patient complained of malaise and loss of appetite while the pyrexia was present, but felt well and wanted to get up during the daily intermissions. The pain in the back remained, and always caused him more trouble than the headache: it was referred to the lumbar spine. Profuse sweats followed the daily pyrexia, and there was a peculiar musty odour, not the sharp acrid smell of acute rheumatism, exhaled from the body.

### History of First Case.

Notes taken on April 18 describe the pupils as rather dilated, equal, and fixed. Tenderness was present in the calves of the legs, along the line of the sciatic nerves, over the erector spinæ muscles, and over the points of exit of the cranial nerves. The abdominal reflex was absent: there was no abdominal tenderness, nor any other abnormality of this region. The knee jerks were exaggerated: knee clonus and ankle clonus were present. No Babinski sign. A modified Kernig's sign was present on both sides, but this might possibly have been due to sciatic tenderness and stretching. Babinski's neck sign was absent; there was no retraction of the head, nor any stiffness of the neck. A sample of blood for culture was taken from the arm, and this was followed by a copious epistaxis, which greatly relieved the headache. On one occasion, two or three days before the date of this particular note, it was thought that a faint systolic apical murmur was present; but it was never detected again, and there was no cardiac abnormality throughout the illness, except that after some weeks the apex beat reached the nipple line in the fifth space, whence it afterwards receded to its previous normal position. The blood was reported to be sterile, and the urine was tested for albumin and sugar with a negative result. The Widal reaction was strongly positive for *Bacillus typhosus*.

The rash was regarded at first as a rheumatic erythema, and salicylates were freely given, but quite without effect. Indeed, the symptoms increased in severity. A note of April 25 states: "Every day, usually in the afternoon, he feels chilly, and the temperature rises rapidly from normal to 103 deg., 104 deg., or 105 deg. Then a profuse sweat breaks out, and for the rest of the twenty-four hours the temperature is normal. Quinine in large doses quite fails to prevent or relieve this symptom. There is no actual rigor." About this time the intermissions of fever became much more definite and regular: the temperature was practically always normal for sixteen or eighteen hours out of the twenty-four, and rose regularly to upwards of 104 at some time or other between 2 p.m. and midnight. During the apyrexial intervals the pulse rate was about 70; even at the height of the fever it only ranged as a rule between 90 and 100. On May 7 the patient's condition was practically unchanged. Another sample of blood was taken and once more found to be sterile. The urine, too, was free from all mobile bacilli. A blood film was stained: no malarial parasites were present, nor were any unusual corpuscles seen in the film. A blood count was not carried out, but there was no obvious excess of leucocytes on the film. The fæces also were cultivated; *B. coli* alone was recovered from them.

On May 13 it is recorded that the man had emaciated a good deal, though he had always taken his (slop) diet well. His complexion was greasy and somewhat hectic. The tongue was still plastered with a whitish fur, but the edges and tip were clean. The left pupil was slightly larger than the right (this observation could never be confirmed subsequently). Both pupils contracted well to light, but on covering the eyes only a minimal amount of dilatation occurred; the pupillary neck reflex was absent. Both knee jerks were exaggerated, and clonus was present on both sides. The triceps jerks were normal. No Babinski reflex. A much nearer approach to Kernig's sign could then be obtained, though not quite a definitely positive one. The abdominal reflex was still absent; the cremasteric reflex was present. Abadie's sign was absent. No anæsthesia was present, except that localisation of tactile sensations on the toes was poor. At the time of making this note (afternoon) the pulse rate was but 52. A blood film was stained for spiral parasites, but none were seen. On the next day, May 14, a fresh crop of erythema appeared; the pupils were equal and reacted well to light and to darkness, but the sympathetic reflex was still absent. From this time onwards a gradual improvement set in. A note of May 19 states: "During the last few days the patient has been decidedly better. The temperature reached 103 deg. on May 17, but has ranged lower than hitherto. Complains now of pain in the back and in the calves of the legs. Both the thighs and the legs are much wasted. The knee jerks are exaggerated, and there is pseudo-clonus of the ankles. There have been no fresh spots for three or four days: the last crop are almost faded. The tongue is still furred, but less so than formerly."

On May 21 a new crop of rash (the last) came out, and the temperature—which had been almost down to normal—rose to 102 deg. The urine was sterile, and the fæces contained no bacilli of the enteric group. A strongly positive Widal reaction was present for *Bacillus typhosus*. From this time onwards the patient did well; from the 26th onwards the temperature was normal and the symptoms all subsided, and on June 2 he was allowed up, with knee jerks and plantar reflexes normal. He convalesced rapidly, considering the severity of his illness, and was transferred convalescent to England on June 13. There was never throughout the case any vomiting, any external ocular palsy, any petechial rash, any stiffness or rigidity of the neck; and though headache was complained of for a few days, it was not very severe. Lumbar puncture was discussed, but it was thought that the indications for it were insufficient. The diagnosis remained a mystery; cerebro-spinal fever was suggested as a possibility, but was rejected by those who had had actual experience of that disease in England.

*(To be continued.)*

